# Out-of-pocket costs near end of life in low- and middle-income countries: A systematic review

**DOI:** 10.1371/journal.pgph.0000005

**Published:** 2022-01-06

**Authors:** Eleanor Reid, Arunangshu Ghoshal, Aisha Khalil, Jingjing Jiang, Charles Normand, Alexandria Brackett, Peter May

**Affiliations:** 1 Department of Emergency Medicine, Yale University School of Medicine, New Haven, Connecticut, United States of America; 2 Usher Institute, University of Edinburgh, Edinburgh, Scotland; 3 Tata Memorial Hospital, Homi Bhabha National Institute, Mumbai, India; 4 INFAQ Foundation, Karachi, Pakistan; 5 Centre for Health Policy and Management, Trinity College Dublin, Dublin, Ireland; 6 Cicely Saunders Institute, King’s College London, London, United Kingdom; 7 Cushing/Whitney Medical Library, Yale University, New Haven, Connecticut, United States of America; 8 The Irish Longitudinal Study on Ageing, Trinity College Dublin, Dublin, Ireland; African Population and Health Research Center, KENYA

## Abstract

**Background:**

Globally, there is a rise in chronic disease, including cancer, major organ failure and dementias. Patients and their families in low- and middle-income countries (LMICs) pay a high proportion of medical costs out of pocket (OOP), and a diagnosis of serious illness often has catastrophic financial consequences. We therefore conducted a review of the literature to establish what is known about OOP costs near end of life in LMICs.

**Aims:**

To identify, organise and report the evidence on out-of-pocket costs in adult end-of-life populations in LMIC.

**Methods:**

A systematic search of 8 databases and a hand search of relevant systematic reviews and grey literature was performed. Two independent reviewers screened titles and abstracts, assessed papers for eligibility and extracted data. The review was registered with PROSPERO and adhered to the Preferred Reporting items for Systematic Reviews and Meta Analyses. The Mixed Methods Appraisal Tool was used to assess quality. The Wagstaff taxonomy was used to describe OOP.

**Results:**

After deduplication, 9,343 studies were screened, of which 51 were read and rejected as full texts, and 12 were included in the final review. OOP costs increased with advanced illness and disease severity. The main drivers of OOP were medications and hospitalizations, with high but variable percentages of the affected populations reporting financial catastrophe, lost income, foregone education and other pressures.

**Conclusion:**

Despite a small number of included studies and heterogeneity in methodology and reporting, it is clear that OOP costs for care near end of life in LMIC represent an important source of catastrophic health expenditures and impoverishment. This suggests a role for widespread, targeted efforts to avoid poverty traps. Financial protection policies for those suffering from incurable disease and future research on the macro- and micro- economics of palliative care delivery in LMIC are greatly needed.

## Introduction

### Background

Low- and middle-income countries (LMICs) face sharply increasing incidence of non-communicable diseases such as cancer, major organ failure, and Alzheimer’s disease and related dementias [1]. Health systems widely lack capacity and resources to meet even current levels of need with curative and supportive treatment [2]. Patients and their families in LMICs pay a high proportion of costs out of pocket, and a diagnosis of serious illness often has catastrophic financial consequences for a household [3].

The end-of-life phase has unique physical, psychological and spiritual challenges [4]. There is also a widely documented association between the last year of life and health care utilization [5], particularly when people die with multiple serious chronic diseases [6]. Therefore end-of-life care brings specific financial pressures for patients and their families in terms of medical care (e.g. paying for medications) and unpaid care, foregone income, and transport to and from appointments [7].

People with terminal illness and their families in LMICs are vulnerable to bankruptcy and impoverishment as populations age, and the poorest sections of society are routinely the most vulnerable of all [8]. One particular susceptibility is spending on supposedly disease-modifying treatments, particularly in the absence of palliative care services that aim to guide the patient and family towards better choices. The downstream effects of palliative care are both improved patient reported outcomes, and cost savings [9, 10]. In LMICs, it is unknown what percentage of the high costs of medical care are borne early versus later on in the disease trajectory: it may be that early drivers of financial hardship begin at time of diagnosis, such that by the time a patient reaches end stage disease, the family has already been burdened by heavy costs. For this reason, in high income settings, there is evidence to support early initiation of palliative care, alongside curative treatments [11, 12].

Faced with impossible choices between earning income or caring for a loved one, or between retaining basic assets or paying for treatment, end-of-life care is a potential poverty trap for many [9]. Palliative care services in those countries are generally underdeveloped with widespread prevalence of unmet need [10].

Out-of-pocket costs in LMICs are a long-standing policy question [3]. The economics of end-of-life care has received growing attention in the last decade, but with a heavy focus on high-income countries where high end-of-life costs for the health system often represent overtreatment and poorly coordinated care between hospital and home settings [13]. The situation in LMICs is fundamentally different: high costs near end of life are widely borne by patients and their families, where the alternative is to receive no treatment [9]. Studies examining out-of-pocket costs and family burden have also dealt predominantly with high-income country settings [14].

### Rationale and aim

We are unaware of any prior review examining the costs to patients and families in the end-of-life phase in LMICs. We therefore conduct a review of the literature to establish what is known about out-of-pocket costs near end of life in those settings. Arising results can inform efforts in low-resource settings to upscale palliative care provision, to design financial protection policies for people with non-communicable disease, and to conduct future research on the economics of palliative and end-of-life care in settings where prior attention has been minimal.

## Materials & methods

### Protocol and registration

We registered a protocol for this systematic review on PROSPERO (CRD42020215188). Registration date was November 19^th^, 2020.

### Search strategy and information sources

A clinical librarian (AB) developed the search strategy after a consultation with ER and PM. AB also received related articles which helped formulate the search strategy with the use of the Yale MeSH Analyzer and were later used to validate search concepts.

The search strategy was peer-reviewed by another senior librarian. The search strategy used both keywords and controlled and indexed vocabulary combining the terms for low-and-middle income countries, cost, and palliative care.

The databases were searched from inception to August 5, 2020; the databases included: MEDLINE (Ovid), Embase (Ovid), APA PsycInfo (Ovid), Global Health (Ovid), CINAHL (Ebsco) Web of Science (indexes: SCI-EXPANDED, SSCI, A&HCI, CPCI-S, CPCI-SSH, BKCI-S, BKCI-SSH, ESCI, CCR-EXPANDED, IC), Scopus, EconLit (ProQuest), and Cochrane CENTRAL. See S1 File for the search details for each database.

We supplemented database searches with other methods. One reviewer (JJ) hand-searched three potentially relevant systematic reviews [10, 15, 16]. One reviewer (AG or AK) hand-searched from 2010 to August 2020 the following journals: Lancet Global Health, BMJ Global Health and Indian Journal of Palliative Care. One reviewer (JJ) hand-searched the following websites as grey literature sources: World Bank, World Health Organization and United Nations University economics department [17–19].

### Eligibility criteria and study selection

#### Inclusion criteria

*Population*. Adults (aged 18+) at the end of life or diagnosed with one of the following serious life-limiting medical illnesses: advanced cancer, serious heart disease (e.g., heart attack, PVD, CHF), major organ failure (e.g., lung, liver, kidney), advanced Alzheimer’s disease and related dementias, AIDS/HIV.

*Intervention*. Palliative care, supportive care and end of life, opioids, and other pain management medications.

*Comparison*. Any or none.

*Outcome*. Out-of-pocket costs incurred by patients and their families in care and treatment for end-of-life care. We include studies that measure the monetary cost of providing informal care, including costed dedicated time, income foregone through lost work, and additional costs incurred (e.g., travel, patient medications).

*Setting*. Low- and middle-income countries, as defined by the World Bank [20].

*Study design*. Any.

#### Exclusion criteria

*Population*. Studies of children. Studies of people with other chronic diseases (e.g., TB, diabetes) as a primary diagnosis. Studies of people in the early stages of a terminal disease.

*Intervention*. Screening, identification, diagnosis of disease. Medications with a predominantly curative intent.

*Outcome*. Costs of care where the patient does not contribute their own resources in money or time, e.g., any service free at the point of use. Prevalence of out-of-pocket payment or unpaid care (e.g., “how many households have an informal carer?”) where that prevalence is not quantified as an estimation of time or cost.

*Setting*. Studies in high-income countries.

*Other*. Studies where our population, outcome and setting are not specifically reported, e.g. (1) an overview of out-of-pocket costs at the population level that does not delineate those with terminal illness or at end of life, e.g. (2) a cost of illness study for a terminal illness that does not delineate out-of-pocket and centrally funded costs. Studies where out-of-pocket costs are hypothetical and/or employed as a predictor (e.g., “Is high potential out-of-pocket costs a reason why women do not attend breast cancer screening?”; “What is the willingness to pay for different cancer treatments?”). Any publication type except research articles (e.g., letters to the editor, conference abstracts). We included only English language articles and retained eligible abstracts in other languages to assess risk of bias from the English-only constraint.

#### Rationale for these criteria

We imposed three basic criteria: LMIC setting, palliative/end-of-life population and out-of-pocket costs as an outcome of interest. Among these, only the first can be categorized discretely according to objective rules (we use The World Bank) [20]. For population, we anticipated challenges in distinguishing the end-of-life phase given that life-limiting illnesses often have poor prognosis in LMIC settings. We did not require that populations are characterized as “end of life” but we did require that the disease be advanced. Populations defined as “early” in a trajectory of serious disease, as well as evaluations of prevention, including vaccination, screening and diagnosis, were excluded. Pharmacoeconomic evaluations of any drugs with life-extending intent were also excluded. For out-of-pocket costs, we defined this broadly to include literal currency spending but also unpaid care and other labor, transport costs, foregone income. Studies that report “total” costs in any circumstance, but do not report separately out-of-pocket and informal care costs, were excluded at full text review. Prior experience showed that evidence on interventions near end of life in LMICs is scant [10], but any study that described, quantified, evaluated determinants, or otherwise addressed specifically our outcome, population and setting of interest were considered eligible.

#### Study selection

Each title/abstract was screened as (in)eligible by two independent reviewers (two of AG, AK, ER, PM). Disagreements were settled by consensus, with either ER or PM acting as the third reviewer.

Full texts were screened using the same process as titles/abstracts. Quality assessment of included studies was performed by two independent reviewers (one of AG and AK, and one of ER and PM) using the MMAT tool. There was no quality cut-off for inclusion, we resolved that eligible studies be reported in the context of their methods and limitations.

### Data extraction and data items

Data were extracted using the same process as titles/abstracts: two independent reviewers, with a third senior reviewer (ER or PM) adjudicating any conflicts. Data were extracted to a bespoke form developed in Excel, that required data points on author, year of publication, country of data collection, year of data collection, study design, sample size (total, exposure, comparison), population, intervention/exposure, comparison, outcome of interest, main results, key themes/messages/strengths, and key limitations. The two reviewers’ outputs were then merged into a single file by a third reviewer (AG or AK), and conflicts or problems solved by consensus.

### Risk of bias within individual studies and across studies

In anticipation of a small, heterogeneous literature we did not evaluate risk of bias specifically but assessed bias as part of quality assessment.

### Summary measures and synthesis of results

In anticipation of a small, heterogeneous literature we did not pre-specify an outcome of interest (e.g., risk ratio or cost-effectiveness ratio) but instead adopted a flexible approach depending on identified studies, which could include descriptive studies. We planned therefore for narrative synthesis, organizing reported out-of-pocket costs according to seven measures in a well-known systematic review: (i) expenditure in absolute (international dollar) terms; (ii) measures of dispersion (or risk); (iii) the out-of-pocket budget share; (iv) progressivity; (v) the incidence of “catastrophic” expenditures; (vi) inequality in the incidence of catastrophic expenditures; (vii) the incidence of “impoverishing” out-of-pocket expenditures, as well as the addition to the poverty gap due to out-of-pocket expenditures [3].

## Results

### Study selection

**Fig 1:**
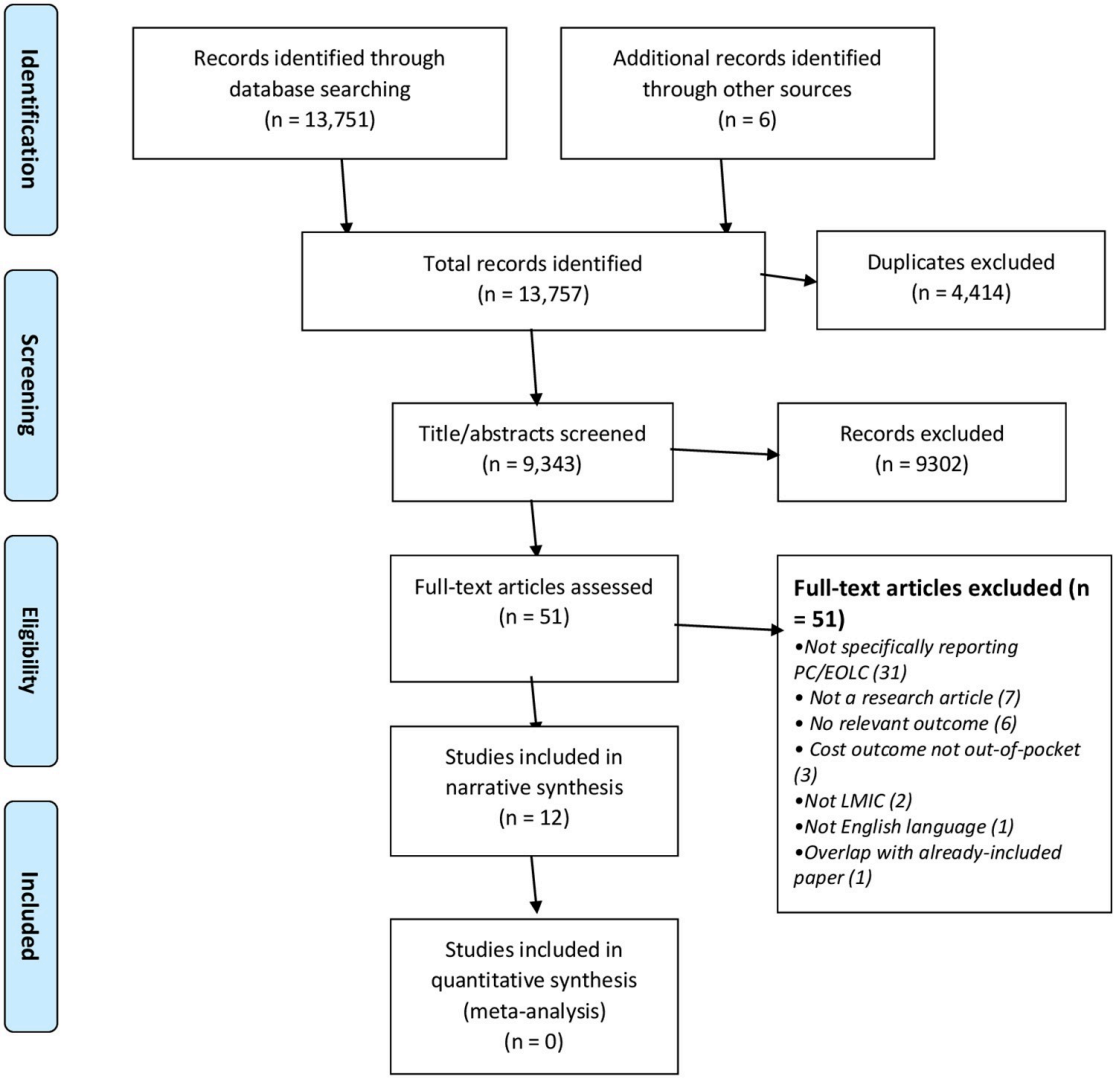
PRISMA diagram.

The database search returned a total of 13,751 records with 9,337 unique articles. The hand search identified six additional studies and the grey literature search identified no relevant studies. These 9,343 studies were screened in Covidence. We excluded 9,280 articles after screening of titles and abstracts, and a further 51 articles after reading the full text. One of these exclusions was due to not being in English [21]. Thus, we included 12 articles in our review. See Fig 1.

### Study characteristics

The data extracted from the twelve included studies [22–33] are presented in Table 1.

**Table 1:** Data extraction- all included studies.

Lead author YearcCountry	Country	Aim(s)	Design	Data sources, years	Inclusion criteria	Sample size	Outcome(s) of interest
Arisar (2020)	Pakistan	To describe the financial burden imposed by chronic liver disease (CLD)	Cross-sectional descriptive prospective study	Primary data collection 2016	CLD patients admitted to a large urban hospital aged 18–80	N = 190, of whom 28 had a severe disease status	Prevalence of objective and subjective financial burden from CLD: savings, paying bills, access to food and medicine, “worsening economic status”
Atieno (2018)	Kenya	To describe the costs of treating cancer patients	Cross-sectional descriptive retrospective study	Secondary analysis of hospital accounting data 2014–16	Random sample of 10% of cancer patients admitted to a large urban hospital aged 18+	N = 412	Direct medical costs of palliative care in KES and USD, “most” paid OOP
Das (2018)	India	To study the trends in out-of-pocket expenditure among hospital inpatients over time, and to compare trends for those dying in hospital and discharged alive	Case-control retrospective study	Secondary analysis of nationally representative survey data 1995–96; 2004–05; 2014–15	Respondents to three waves of a recurring survey of ‘morbidity and health care’	N = 94,084 Decedents (cases): 4,357 Survivors (controls): 89,727	OOP inpatient expenditure in INR
Emanuel (2010)	India	To gather pilot data on the economic impact of terminal illness on families, and on the feasibility of training caregivers as a method of stemming illness-related poverty.	Exploratory, descriptive prospective study	Primary data collection 2008	Convenience sampling of families in contact with a single palliative care provider	N = 11 patient-caregiver dyads (22 participants)	Total debt, total health care spending in INR, USD Prevalence of objective and subjective financial burden: giving up work, missing/ discontinuing education, lost income, forced to sell assets, feeling pressure to marry, feeling pressure to generate other income
Kimman (2015)	Cambodia, Indonesia, Laos, Malaysia, Phillipines, Thailand, Vietnam	To describe prevalence of death and financial catastrophe in the year following a cancer diagnosis	Prospective longitudinal study	Primary data collection 2012–13	People admitted to 47 public and private hospitals and cancer centres with a first-time cancer aged 18+	N = 9,513	Prevalence of financial catastrophe, defined as out-of-pocket medical costs exceeding 30% of annual income
Kongpakwattana (2019)	Thailand	To describe costs, quality of life and caregiver burden associated with Alzheimer’s disease (AD).	Cross-sectional descriptive prospective study	Primary data collection and secondary analysis of hospital accounting data 2017–18	People admitted to a large urban hospital with a diagnosis of AD for at least six months and aged 60+	N = 148, of whom 54 had a severe disease status	Out-of-pocket expenditure in USD, unpaid caregiver burden
Kumar (2018)	India	To describe socio- economic status and demographic profile of patients with advanced cancer receiving palliative care.	Cross-sectional descriptive prospective study	Primary data collection 2017	Patients with advanced cancer receiving outpatient palliative care at a single institution.	N = 80	Subjective financial burden: financial insecurity
Ladusingh (2013)	India	To compare OOP inpatient care expenditures of decedents and survivors	Case-control retrospective study	Secondary analysis of nationally representative survey data 2004–05	Respondents to one wave of a recurring survey of ‘morbidity and health care’	N = 31,868 Decedents (cases): 715 Survivors (controls): 31,153	Out-of-pocket expenditure in INR
Leng (2019)	China	To describe cancer treatments and care costs during the last 3 months of life, and to compare geographical disparities	Cross-sectional descriptive retrospective study	Primary data collection 2016	Caregivers of people who died from cancer between 2013 and 2016.	N = 792	End-of-life (EOL) health care costs in USD; prevalence of borrowing to meet EOL costs
Ngalula (2002)	Tanzania	To assess the levels and determinants of medical care use and household expenditure during terminal illness.	Cross-sectional descriptive retrospective study	Secondary analysis of longitudinal survey data 1996–98	Households of people who had died aged 15–59 with an established cause of death	N = 181	Prevalence of health care expenditure and funeral expenses borne OOP; received financial assistance outside the family; sold property for care or funeral
Prinja (2018)	India	To assess the health system costs, out-of-pocket (OOP) expenditure and extent of financial risk protection associated with treatment of liver disorders.	Case-control retrospective study	Primary data collection and secondary analysis of hospital accounting data 2013–14	Liver disorder patients admitted to the intensive care unit or high dependency unit of a large urban hospital	N = 150	Out-of-pocket expenditure in USD and INR; prevalence of catastrophic health expenditure, defined as out-of-pocket medical costs exceeding 40% of non-subsistence expenditure
Ratcliff (2017)	India	To describe prevalence of household poverty, association between poverty and palliative care receipt	Single cohort descriptive study, interviews conducted once on experiences at different time points	Primary data collection 2015	Patients or family members at one of three rural palliative care providers	N = 129	Prevalence of poverty, medical costs, lost work, debts, sale of assets; association between palliative care receipt and medical costs, work

**Fig 2:**
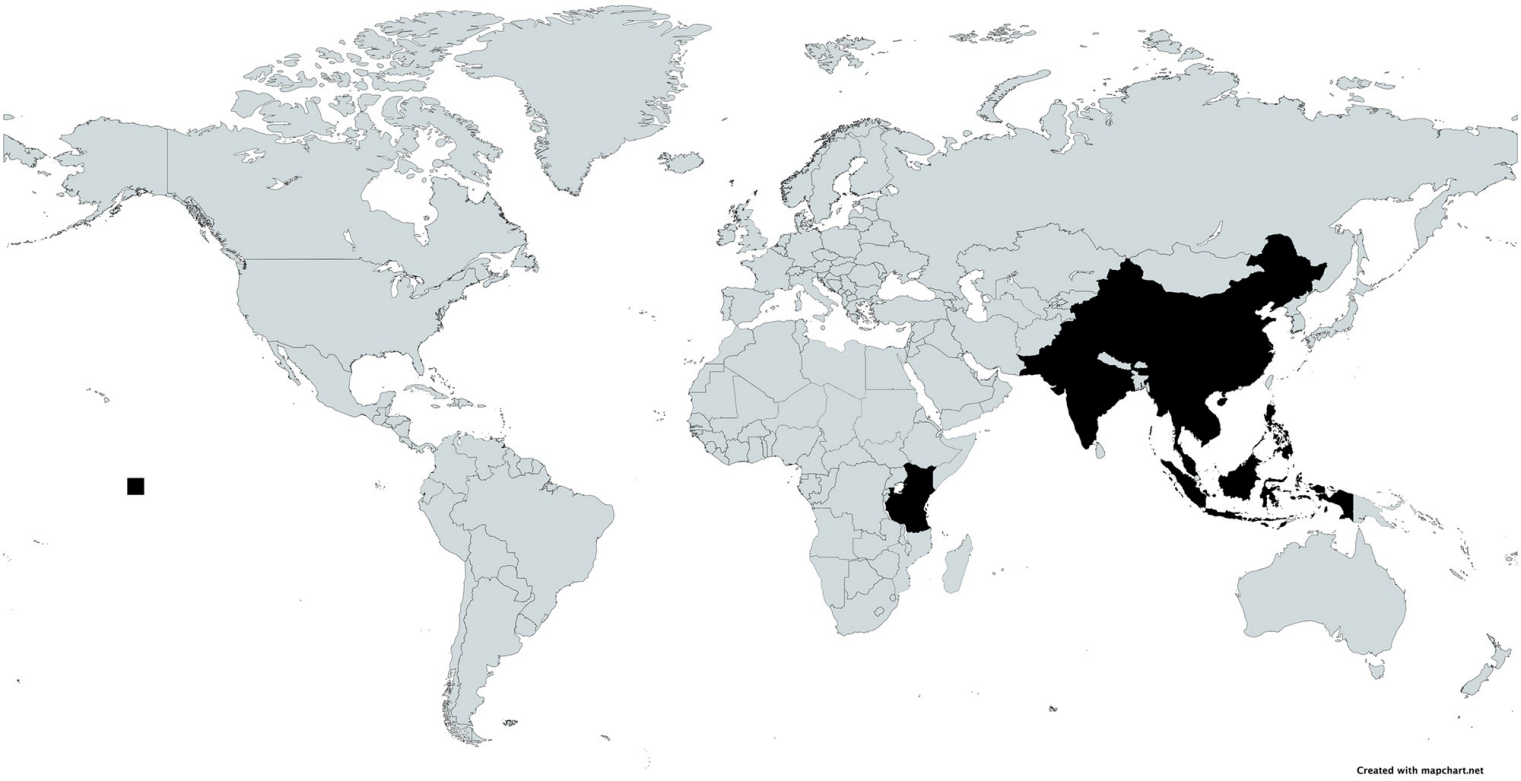
Map of included countries. Black = Country included in systematic review.

The earliest study was published in 2002 and the most recent in 2020 with a median publication year of 2018. There were six studies conducted in India, and one each conducted in China, Kenya, Pakistan, Tanzania, Thailand and Southeast Asia (covering Cambodia, Indonesia, Laos, Malaysia, Philippines, Thailand, and Vietnam). See Fig 2.

There were six descriptive cross-sectional studies, of which three were conducted prospectively [26, 27, 32] and three retrospectively [22, 29, 30]. Three further prospective studies involved original data collection: one longitudinal study,[25] one cohort study [33] and one exploratory pilot study.[24] Three were retrospective case-control studies comparing people who died in hospital with those who did not. [23, 28, 31]. No study evaluated the effects of an intervention. The largest study had 94,084 participants, the smallest study had 11 participants with a median sample size of 185.5.

### Sampling

Six studies based their sampling frames on diagnosis of a specific disease: three in cancer, [22, 25, 29] two in liver disease [31, 32] and one in Alzheimer’s disease [26]. Three studies conducted convenience sampling via a palliative care provider [24, 27, 33]. Two studies analyzed large population-representative surveys on health and morbidity [23, 28]. One study surveyed households that had recently suffered a bereavement [30].

### Outcomes

Nine studies evaluated outcomes specified by the taxonomy of Wagstaff et al [3]. Two studies measured prevalence of financial catastrophe, contextualizing costs according to the means of people with serious illness and their families. [25, 31] Seven studies quantified out-of-pocket spending and/or debt in absolute currency amounts [22–24, 26, 28, 29, 31]. Additionally, one study quantified unpaid carer burden [26] and three studies measured objective and/or subjective financial burden [24, 30, 33].

### Quality of reporting

Quality assessment were performed by two independent reviewers (one of AG and AK, and one of ER and PM) using the MMAT tool. The MMAT is intended to be used as a checklist for concomitantly appraising and/or describing studies included in systematic mixed studies reviews (reviews including original qualitative, quantitative, and mixed methods studies). There was no quality cut-off for inclusion, and studies have been reported in the context of their methods and limitations. A summary of the quality assessments is provided in Table 2. Full details of the MMAT scores are provided as S2 File.

**Table 2:** MMAT quality results.

		Atieno	Arisar	Das	Emanuel	Kimman	Kongpakwattana	Kumar	Ladusingh	Leng	Ngalula	Prinja	Ratcliff
Screening questions	Are there clear research questions?												
	Do the collected data allow to address the research questions?												
Qualitative	1.1. Is the qualitative approach appropriate to answer the research question?												
	1.2. Are the qualitative data collection methods adequate to address the research question?												
	1.3. Are the findings adequately derived from the data?												
	1.4. Is the interpretation of results sufficiently substantiated by data?												
	1.5. Is there coherence between qualitative data sources, collection, analysis and interpretation?												
Quantitative randomized controlled (trials)	2.1. Is randomization appropriately performed?												
	2.2. Are the groups comparable at baseline?												
	2.3. Are there complete outcome data?												
	2.4. Are outcome assessors blinded to the intervention provided?												
	2.5 Did the participants adhere to the assigned intervention?												
Quantitative non-randomized	3.1. Are the participants representative of the target population?												
	3.2. Are measurements appropriate regarding both the outcome and intervention (or exposure)?												
	3.3. Are there complete outcome data?												
	3.4. Are the confounders accounted for in the design and analysis?												
	3.5. During the study period, is the intervention administered (or exposure occurred) as intended?												
Quantitative descriptive	4.1. Is the sampling strategy relevant to address the quantitative research question?												
	4.2. Is the sample representative of the target population?												
	4.3 Are measurements appropriate?												
	4.4 Is the risk of non-response bias low?												
	4.5 Is the statistical analysis appropriate to answer the research question?												
Mixed-methods	5.1. Is there an adequate rationale for using a mixed methods design to address the research question?												
	5.2. Are the different components of the study effectively integrated to answer the research question?												
	5.3. Are the outputs of the integration of qualitative and quantitative components adequately interpreted?												
	5.4. Are divergences and inconsistencies between quantitative and qualitative results adequately addressed?												
	5.5. Do the different components of the study adhere to the quality criteria of each tradition of the methods involved?												

Green = Yes.

Red = No.

Yellow = Unable to determine.

### Main results

Main results of each included study are presented in Tables 3–6, separated by outcome.

**Table 3:** Results of studies quantifying catastrophic financial circumstances.

Study	Scope	Summary statistics	Key conclusions
**Kimman (2015)**	Prevalence of death and financial catastrophe, defined as out-of-pocket medical costs exceeding 30% of annual income, in the year following a cancer diagnosis	Death: 29% Alive financial catastrophe: 48% Alive no financial catastrophe: 23%	•A minority of people experienced neither death nor financial catastrophe in the year following a cancer diagnosis
			•Both death and financial catastrophe were significantly associated with proximity to death and baseline socioeconomic status
			•Insurance was also protective against financial catastrophe
**Prinja (2018)**	Prevalence of death and financial catastrophe, defined as exceeding 40% of non-subsistence expenditure, among hospital inpatients	All, financial catastrophe: 78% Died, financial catastrophe: 92% Alive, financial catastrophe: 75%	•Very high prevalence of financial catastrophe among both decedents and survivors
			•Prevalence higher in intensive care unit among survivors, and higher in high dependency unit among decedents
			•Medicines account substantively for costs, showing that purchasing schemes could reduce financial catastrophe

**Table 4:** Results of studies quantifying out-of-pocket spending, debt.

Study	Scope	Summary statistics	Key conclusions
**Emanuel (2010)**	Total OOP spending on health care last two months, total debt among people receiving palliative care	Mean total spending: INR 15,000 (USD 325) Mean debt: INR 50,000 (USD 1,082)	•High prevalence of debt, out-of-pocket spending
**Ladusingh (2013)**	Total OOP spending among hospital inpatients	Mean costs Decedents (2004/5): INR 10,036 Survivors (2004/5): INR 7,126	•OOP spending was significantly higher for those dying in hospital than those discharged alive
**Atieno (2018)**	Total direct medical costs of palliative care in people with cancer	Average cost of palliative care: KES 98,931 (USD 977) Sub-group in public hospital: KES 94,867 (USD 937) Sub-group in private hospital: KES 118,708 (USD 1172)	•Wide variation in cancer costs by diagnosis and treatment choices •Palliative care accounted for 12% of cancer costs •Other cost categories, e.g. surgery, chemotherapy, were not reported specifically for end-of-life population
**Das (2018)**	Total OOP spending among hospital inpatients	Mean costs Decedents (2004/5): INR 22,649 Survivors (2004/5): INR 15,485 Decedents (2014/5): INR 43,897 Survivors (2014/5): INR 19,438	•OOP spending was significantly higher for those dying in hospital than those discharged alive •Mean decedent OOP spending nearly doubled during the ten-year observation period
**Prinja (2018)**	Total OOP spending among hospital inpatients with liver disease	Mean costs All (ICU): INR 2,465 Decedents (ICU): INR 2,128 Survivors (ICU): INR 2,465 All (ICU): INR 1,752 Decedents (ICU): INR 3,553 Survivors (ICU): INR 1,484	•OOP costs are higher in intensive care unit among survivors, and higher in high dependency unit among decedents •Wide variation in costs by locality, education, occupation, but these difference are not statistically significant
**Kongpakwattana (2019)**	Total OOP spending among people diagnosed with dementia	Mean OOP spending Mild: USD 509 Moderate: USD 951 Severe: USD 1003	•Mean OOP spending was highest among the severe disease group, but this difference was not statistically significant
**Leng (2019)**	Total end-of-life (EOL) health care costs among cancer decedents	Mean total spending OOP: USD 11,288	•OOP spending was 36% of total cancer costs •OOP spending was significantly higher in urban areas than rural, geographic variation was also identified

**Table 5:** Results of studies quantifying unpaid care.

Study	Scope	Summary statistics	Key conclusions
**Kongpakwattana (2019)**	Hours’ unpaid caregiving per month for people diagnosed with dementia	Mean total caregiving time Mild: 204 hours Moderate: 253 hours Severe: 354 hours	•Unpaid caregiving was highest among the severe disease group, and this difference was statistically significant

**Table 6:** Results of studies reporting prevalence of objective and subjective financial burden.

Study	Scope	Summary statistics	Key conclusions
**Ngalula (2002)**	Among people who died, prevalence of health care expenditure and funeral expenses borne by decedent and spouse; received financial assistance outside the family; sold property for care or funeral	Decedent or spouse was primary source of payment: AIDS: 37% Other diseases: 48% Injuries: 14% Received financial assistance outside the family: AIDS: 1% Other diseases: 5% Injuries: 0% Sold property for care or funeral: AIDS: 29% Other diseases: 10% Injuries: 19%	•Widespread prevalence of decedents and families meeting costs, including selling property •Little evidence of financial assistance outside the family
**Emanuel (2010)**	Prevalence of objective and subjective financial burden among people receiving palliative care	Patient left workforce: 100% Family members working less: 100% Education missed or foregone: 100% Had to sell assets: 100% Married/feeling pressure to marry: 45% Resort to dangerous or illegal work: 64%	•Foregone work, income, education reported by all participants •Widespread prevalence of pressure to replace lost income and meet medical costs
**Ratcliff (2017)**	Prevalence of household poverty, association between poverty and palliative care receipt	Patient left workforce: 66% Family member left workforce: 26% In debt: 98% Had to sell assets: 59%	•Widespread prevalence of lost income, debt, pressure to sell assets •Palliative care reportedly associated with lower out-of-pocket spending and high income through return to work and awareness of government benefits. No formal statistical evaluation of this.
**Kumar (2018)**	Prevalence of subjective financial burden among patients with advanced cancer receiving palliative care	Financial insecurity among participants: 26% Concern regarding financial security of family members in future: 33%	•Widespread prevalence of insecurity about current and future finances
**Leng (2019)**	Prevalence of borrowing among cancer decedents	Borrowing to meet EOL expenses: 33%	•Widespread prevalence of borrowing, with variation by urban/rural, geographic region and spending type
**Arisar (2020)**	Prevalence of objective and subjective financial burden among patients with severe chronic liver disease	Worsening economic status: 89% Stopped saving: 89% Utilization of previous savings: 93% Need to borrow to meet expenses: 71% Delay in repaying loans: 36% Delay in children’s education: 61% Electricity/gas cut off: 14% Had to leave current house: 14% Had to forego food: 22% Unpaid medical expenses: 47% Skip medicines: 72% Buy fewer medicines than required: 61% Missed surgery: 57% Missed doctor’s appointment: 61%	•Socioeconomic burden of chronic liver disease is considerable •Magnitude of burden is strongly associated with severity of disease–and is therefore greatest in the end-of-life cohort

### Synthesis of results

#### Financial catastrophe

Results with respect to financial catastrophe are summarized in Table 3.

In India Prinja et al. [31] reported that 92% of people who died in hospital with advanced liver disease suffered catastrophic finances, defined as out-of-pocket spending that exceeded 40% of subsistence income. Medicines accounted substantively for costs, meaning that publicly funded purchasing schemes could address directly this financial catastrophe.

Kimman et al. [25] followed people for a year following a cancer diagnosis in seven South East Asian countries and found that only 23% were both alive and free of financial catastrophe, defined as out-of-pocket medical costs exceeding 30% of annual income. Among the sample, 48% were alive but facing financial catastrophe, and 29% had died. Since death and financial catastrophe are reported as competing outcomes, it is not known what proportion of those who died experienced high OOP spending and as such the results are not directly comparable with those of Prinja et al [31].

#### Out-of-pocket costs in absolute terms

Results with respect to costs in absolute terms are summarized in Table 4. All studies reported substantial costs for their populations of interest, and additional factors that determined or mediated costs were identified.

Three studies in the hospital setting found that OOP costs for deceased patients in inpatient care is much greater than that of survivors [23, 28, 31]. Moreover, both total costs for decedents and the difference between decedent and survivor costs appear to be growing over time [21].

Cancer costs in private health care settings were higher than those in public settings [20], and higher for patients living in urban areas than rural areas [29]. People already receiving palliative care have high levels of cost and associated debt, [24] and magnitude of costs is strongly associated with disease severity [26, 32].

#### Unpaid carer burden

One study looked at unpaid carer burden, summarized in Table 5 [26]. Unpaid caregiving hours were high in all stages of Alzheimer’s disease and highest among the severe disease group. This difference was statistically significant.

#### Objective and subjective financial burden

Studies quantifying the incidence or prevalence of financial burden in descriptive measures are summarized in Table 6.

These measures affirm the findings in other tables that advanced medical illness results in high costs and financial pressures. Further, illness and associated costs catalyze vicious economic circles for patients and families. Persons with serious illness overwhelmingly lose their jobs or leave the workforce [24, 33]. Family members leave their jobs and drop out of education to provide care [24, 32]. To meet costs, patients and their families borrow money [29, 32, 33] and sell assets, [24, 30, 33] and in some cases forego food, medicines and health care. [32] Financial pressures confer anxiety about the household’s future [27]. Family members feel pressure to marry, or to engage in risky or illegal activity, in order to address lost income and rising costs [24].

## Discussion

### Key results

Patients approaching end of life in LMIC pay a high proportion of medical costs out of pocket and often suffer catastrophic financial consequences, yet there is a dearth of robust, large scale health economic data to guide hypothesis-generating, poverty-reducing solutions. Financial catastrophe from serious medical illness is not only an issue for patients and families in the end-of-life stage but creates cyclical poverty traps: lost income, increasing borrowing and debt, anxiety and insecurity, and growing pressure to address financial pressures through drastic measures. Our systematic review directly addresses this global inequity with a rigorous search and summary of the existing literature, including a quality assessment.

### Strengths and limitations

Strengths of the review include the broad search terms, inclusion of grey literature, the geographical heterogeneity in the included studies country of origin, and use of two independent reviewers at each step of the review. Limitations of this review are that much of the existing literature is from household surveys and interviews, thus introducing bias however this is mitigated by country-specific estimates for OOP which were included in three of our included studies. Only studies in English were included.

### Interpretation

Inadequate government spending on health is a recurring feature in LMICs. Weak health infrastructure leads to delays in diagnosis and resulting late disease presentations, heavy reliance on out-of-pocket payments and catastrophic health expenditures. Those patients impacted the most include those from remote areas, those with longer and repeat hospitalizations and with the following diagnoses: cancer, Alzheimer’s disease, terminal HIV/AIDs and end stage liver disease. As out-of-pocket spending is inversely proportional to life expectancy, earlier diagnoses through improved screening programs would likely result in more treatable disease, at lower cost.

Policies aimed at bolstering socioeconomic resilience and financial protection are greatly needed in LMICs. A better understanding of early versus late drivers of medical impoverishment is an urgent research priority, as this would inform these strategies. In addition to earlier detection of disease, the provision of early, home-based and widespread access to palliative care in LMIC would serve to decrease OOP and CHE for those with incurable disease, and thus should be considered as a critical health priority and global poverty-reduction strategy. Furthermore, in resource-scare environments, increased access to palliative care would have the secondary effect of liberating limited health resources for those patients with curable disease thus benefiting society at large. The need for greater access to palliative care in LMICs is clear and the evidence for its role as a poverty reduction strategy is emerging. Future research should focus on the implementation and health economic outcomes of palliative care in LMICs, thus improving care for billions of the most vulnerable patients in our global population.

Globally, demographic ageing is likely to result in an increased burden on all levels of health systems and household services, partly due to the relatively long duration of illness at the end of life. Holistic palliative care can mitigate the desperate poverty caused by life-limiting illness, particularly if initiated early in the illness, on a regular basis and on a broad scale. Home-based treatment also frees up hospitals to serve patients with reversible conditions.

## Conclusion

Patients approaching end of life in LMIC pay a high proportion of medical costs out of pocket and often suffer financial catastrophe. These effects are long term and potential poverty traps: reduced household income, rising debt, deteriorating mental health, and narrowing life choices. Policies and interventions are needed to prevent these often-avoidable crises. Evidence to inform such policies and interventions is currently thin.

## Supporting information

S1 Checklist (DOCX)

S1 File Search strategy. A text file of the search strategies used in our systematic review.
(DOCX)

S2 File MMAT. Full details of the MMAT scores which are summarized in Table 2.
(XLSX)
