# Supplementary material for: Out-of-pocket costs near end of life in low- and middle-income countries: A systematic review
Source: PLOS Glob Public Health. 2022 Jan 6;2(1):e0000005. doi: 10.1371/journal.pgph.0000005 (PMC10022295; doi:10.1371/journal.pgph.0000005)
Supplement: S1 File — A text file of the search strategies used in our systematic review. (DOCX) [file pgph.0000005.s002.docx]

Search log, August 5, 2020

Database: Ovid MEDLINE(R) ALL <1946 to August 03, 2020>

Search Strategy:

--------------------------------------------------------------------------------

1 (lmic or lmics or lami countr* or lic or lics).ti,ab. (6456)

2 ((developing or under developed or under-developed or underdeveloped or middle income or low* income or less* developed or under served or under-served or underserved or poor* or deprived) adj3 (countr* or world or population* or nation* or gross domestic or gross national or gdp or gmp or economics or economy)).tw,kw. (112972)

3 exp Developing Countries/ (74836)

4 (Afghanistan or Guinea-Bissau or Sierra Leone or Burkina Faso or Haiti or Somalia or Burundi or Dem* People* Rep* Korea or North Korea or PRK South Sudan or Central African Republic or Liberia or Sudan or Chad or Madagascar or Syrian Arab Republic or Congo or Malawi or Tajikistan or Eritrea or Mali or Togo or Ethiopia or Mozambique or Uganda or Gambia or Niger or Yemen or Guinea or Rwanda).tw,kw. (208656)

5 (Angola or Honduras or Papua New Guinea or Algeria or India or Philippines or Bangladesh or Kenya or (Sao Tome and Principe) or Benin or Kiribati or Senegal or Bhutan or Kyrgyz Republic or Solomon Islands or Bolivia or Lao PDR or Sri Lanka or Cabo Verde or Lesotho or Tanzania or Cambodia or Mauritania or Timor-Leste or Cameroon or Micronesia or Tunisia or Comoros or Moldova or Ukraine or Congo or Mongolia or Uzbekistan or Cote d$Ivoire or Morocco or Vanuatu or Djibouti or Myanmar or Vietnam or (Egypt and Arab) or Nepal or West Bank or Gaza or El Salvador or Nicaragua or Zambia or Eswatini or Nigeria or Zimbabwe or Ghana or Pakistan).tw,kw. (310146)

6 or/1-5 (608627)

7 exp Financing, Personal/ (6075)

8 exp "Cost of Illness"/ (27299)

9 exp "Costs and Cost Analysis"/ (237294)

10 exp Palliative Care/ec [Economics] (1086)

11 exp Caregivers/ec [Economics] (1010)

12 exp Health Expenditures/ (22271)

13 ((personal* or individual* or household* or caregiver* or out-of-pocket* or out of pocket) adj3 (cost or costs or expense or expenditure* or finance or financial or financing or incurred or payment*)).tw,kw. (12987)

14 ((economic or bankruptcy or poverty) adj3 (risk* or burden)).tw,kw. (13606)

15 or/7-14 (257603)

16 exp Palliative Care/ (54128)

17 exp Terminal Care/ (51274)

18 exp Hospices/ (5024)

19 exp "Aged, 80 and over"/ (914192)

20 (palliative or end of life or end-of-life or (end* adj3 life) or terminal or death or dying or mortality or incurable or futile or life limit* or life-limit* or end stage or end-stage or advanced stage* or advanced-stage* or serious illness or hospice*).tw,kw. (1892603)

21 or/16-20 (2694397)

22 6 and 15 and 21 (2872)

Database: Embase <1974 to 2020 August 04>

Search Strategy:

--------------------------------------------------------------------------------

1 (lmic or lmics or lami countr* or lic or lics).ti,ab. (8274)

2 ((developing or under developed or under-developed or underdeveloped or middle income or low* income or less* developed or under served or under-served or underserved or poor* or deprived) adj3 (countr* or world or population* or nation* or gross domestic or gross national or gdp or gmp or economics or economy)).tw,kw. (147655)

3 exp developing country/ (94004)

4 (Afghanistan or Guinea-Bissau or Sierra Leone or Burkina Faso or Haiti or Somalia or Burundi or Dem* People* Rep* Korea or North Korea or PRK South Sudan or Central African Republic or Liberia or Sudan or Chad or Madagascar or Syrian Arab Republic or Congo or Malawi or Tajikistan or Eritrea or Mali or Togo or Ethiopia or Mozambique or Uganda or Gambia or Niger or Yemen or Guinea or Rwanda).tw,kw. (223142)

5 (Angola or Honduras or Papua New Guinea or Algeria or India or Philippines or Bangladesh or Kenya or (Sao Tome and Principe) or Benin or Kiribati or Senegal or Bhutan or Kyrgyz Republic or Solomon Islands or Bolivia or Lao PDR or Sri Lanka or Cabo Verde or Lesotho or Tanzania or Cambodia or Mauritania or Timor-Leste or Cameroon or Micronesia or Tunisia or Comoros or Moldova or Ukraine or Congo or Mongolia or Uzbekistan or Cote d$Ivoire or Morocco or Vanuatu or Djibouti or Myanmar or Vietnam or (Egypt and Arab) or Nepal or West Bank or Gaza or El Salvador or Nicaragua or Zambia or Eswatini or Nigeria or Zimbabwe or Ghana or Pakistan).tw,kw. (394737)

6 or/1-5 (736007)

7 (exp financial management/ or exp "health care cost"/) and personal.ti,ab. (11558)

8 exp "cost of illness"/ (19265)

9 exp "cost benefit analysis"/ (84730)

10 exp palliative therapy/ and (exp economics/ or exp health economics/) (7366)

11 exp caregiver/ and (exp economics/ or exp health economics/) (8552)

12 exp "health care cost"/ (291918)

13 ((personal* or individual* or household* or caregiver* or out-of-pocket* or out of pocket) adj3 (cost or costs or expense or expenditure* or finance or financial or financing or incurred or payment*)).tw,kw. (17552)

14 ((economic or bankruptcy or poverty) adj3 (risk* or burden)).tw,kw. (22118)

15 or/7-14 (405467)

16 exp palliative therapy/ (110752)

17 exp Terminal Care/ (69149)

18 exp hospice/ (13379)

19 exp frail elderly/ or exp very elderly/ (208348)

20 (palliative or end of life or end-of-life or (end* adj3 life) or terminal or death or dying or mortality or incurable or futile or life limit* or life-limit* or end stage or end-stage or advanced stage* or advanced-stage* or serious illness or hospice*).tw,kw. (2613579)

21 or/16-20 (2842481)

22 6 and 15 and 21 (4316)

Database: APA PsycInfo <1806 to July Week 4 2020>

Search Strategy:

--------------------------------------------------------------------------------

1 (lmic or lmics or lami countr* or lic or lics).mp. (1086)

2 ((developing or under developed or under-developed or underdeveloped or middle income or low* income or less* developed or under served or under-served or underserved or poor* or deprived) adj3 (countr* or world or population* or nation* or gross domestic or gross national or gdp or gmp or economics or economy)).mp. (23363)

3 exp Developing Countries/ (5515)

4 (Afghanistan or Guinea-Bissau or Sierra Leone or Burkina Faso or Haiti or Somalia or Burundi or Dem* People* Rep* Korea or North Korea or PRK South Sudan or Central African Republic or Liberia or Sudan or Chad or Madagascar or Syrian Arab Republic or Congo or Malawi or Tajikistan or Eritrea or Mali or Togo or Ethiopia or Mozambique or Uganda or Gambia or Niger or Yemen or Guinea or Rwanda).mp. (20202)

5 (Angola or Honduras or Papua New Guinea or Algeria or India or Philippines or Bangladesh or Kenya or (Sao Tome and Principe) or Benin or Kiribati or Senegal or Bhutan or Kyrgyz Republic or Solomon Islands or Bolivia or Lao PDR or Sri Lanka or Cabo Verde or Lesotho or Tanzania or Cambodia or Mauritania or Timor-Leste or Cameroon or Micronesia or Tunisia or Comoros or Moldova or Ukraine or Congo or Mongolia or Uzbekistan or Cote d$Ivoire or Morocco or Vanuatu or Djibouti or Myanmar or Vietnam or (Egypt and Arab) or Nepal or West Bank or Gaza or El Salvador or Nicaragua or Zambia or Eswatini or Nigeria or Zimbabwe or Ghana or Pakistan).mp. (60228)

6 or/1-5 (94308)

7 exp finance/ and personal.ti,ab. (990)

8 exp Health Care Costs/ (22023)

9 exp "Costs and Cost Analysis"/ (42374)

10 exp Palliative Care/ and exp Economics/ (202)

11 exp Caregivers/ and exp Economics/ (428)

12 ((personal* or individual* or household* or caregiver* or out-of-pocket* or out of pocket) adj3 (cost or costs or expense or expenditure* or finance or financial or financing or incurred or payment*)).mp. (5495)

13 ((economic or bankruptcy or poverty) adj3 (risk* or burden)).mp. (4207)

14 or/7-13 (50894)

15 exp Palliative Care/ (14054)

16 exp Hospice/ (3252)

17 exp geriatric patients/ (13408)

18 (palliative or end of life or end-of-life or (end* adj3 life) or terminal or death or dying or mortality or incurable or futile or life limit* or life-limit* or end stage or end-stage or advanced stage* or advanced-stage* or serious illness or hospice*).mp. (155966)

19 or/15-18 (168516)

20 6 and 14 and 19 (236)

Database: Global Health <1910 to 2020 Week 30>

Search Strategy:

--------------------------------------------------------------------------------

1 (lmic or lmics or lami countr* or lic or lics).ti,ab. (2420)

2 ((developing or under developed or under-developed or underdeveloped or middle income or low* income or less* developed or under served or under-served or underserved or poor* or deprived) adj3 (countr* or world or population* or nation* or gross domestic or gross national or gdp or gmp or economics or economy)).af. (1017907)

3 Developing Countries/ (999084)

4 (Afghanistan or Guinea-Bissau or Sierra Leone or Burkina Faso or Haiti or Somalia or Burundi or Dem* People* Rep* Korea or North Korea or PRK South Sudan or Central African Republic or Liberia or Sudan or Chad or Madagascar or Syrian Arab Republic or Congo or Malawi or Tajikistan or Eritrea or Mali or Togo or Ethiopia or Mozambique or Uganda or Gambia or Niger or Yemen or Guinea or Rwanda).ti,ab. (105115)

5 (Angola or Honduras or Papua New Guinea or Algeria or India or Philippines or Bangladesh or Kenya or (Sao Tome and Principe) or Benin or Kiribati or Senegal or Bhutan or Kyrgyz Republic or Solomon Islands or Bolivia or Lao PDR or Sri Lanka or Cabo Verde or Lesotho or Tanzania or Cambodia or Mauritania or Timor-Leste or Cameroon or Micronesia or Tunisia or Comoros or Moldova or Ukraine or Congo or Mongolia or Uzbekistan or Cote d$Ivoire or Morocco or Vanuatu or Djibouti or Myanmar or Vietnam or (Egypt and Arab) or Nepal or West Bank or Gaza or El Salvador or Nicaragua or Zambia or Eswatini or Nigeria or Zimbabwe or Ghana or Pakistan).ti,ab. (252907)

6 or/1-5 (1055212)

7 exp finance/ and personal.ti,ab. (51)

8 health care costs/ and personal.ti,ab. (225)

9 ((personal* or individual* or household* or caregiver* or out-of-pocket* or out of pocket) adj3 (cost or costs or expense or expenditure* or finance or financial or financing or incurred or payment*)).af. (5419)

10 ((economic or bankruptcy or poverty) adj3 (risk* or burden)).af. (5945)

11 or/7-10 (11243)

12 exp hospices/ (230)

13 exp hospice care/ (210)

14 (palliative or end of life or end-of-life or (end* adj3 life) or terminal or death or dying or mortality or incurable or futile or life limit* or life-limit* or end stage or end-stage or advanced stage* or advanced-stage* or serious illness or hospice*).af. (338897)

15 or/12-14 (338897)

16 6 and 11 and 15 (932)

CINAHL (Ebsco)—2,830 results

S7 S1 AND S5 AND S6 2,830

S6 MH "Developing Countries" OR TI ( lmic or lmics or lami countr* or lic or lics ) OR AB ( lmic or lmics or lami countr* or lic or lics ) OR SU ( (developing or "under developed" or under-developed or underdeveloped or "middle income" or "low income" or "less developed" or "under served" or under-served or underserved or poor* or deprived) N3 (countr* or world or population* or nation* or gross domestic or gross national or gdp or gmp or economics or economy) ) OR TI ( Afghanistan or Guinea-Bissau or Sierra Leone or Burkina Faso or Haiti or Somalia or Burundi or Dem* People* Rep* Korea or North Korea or PRK South Sudan or Central African Republic or Liberia or Sudan or Chad or Madagascar or Syrian Arab Republic or Congo or Malawi or Tajikistan or Eritrea or Mali or Togo or Ethiopia or Mozambique or Uganda or Gambia or Niger or Yemen or Guinea or Rwanda ) OR AB ( Afghanistan or Guinea-Bissau or Sierra Leone or Burkina Faso or Haiti or Somalia or Burundi or Dem* People* Rep* Korea or North Korea or PRK South Sudan or Central African Republic or Liberia or Sudan or Chad or Madagascar or Syrian Arab Republic or Congo or Malawi or Tajikistan or Eritrea or Mali or Togo or Ethiopia or Mozambique or Uganda or Gambia or Niger or Yemen or Guinea or Rwanda ) OR TI ( Angola or Honduras or Papua New Guinea or Algeria or India or Philippines or Bangladesh or Kenya or (Sao Tome and Principe) or Benin or Kiribati or Senegal or Bhutan or Kyrgyz Republic or Solomon Islands or Bolivia or Lao PDR or Sri Lanka or Cabo Verde or Lesotho or Tanzania or Cambodia or Mauritania or Timor-Leste or Cameroon or Micronesia or Tunisia or Comoros or Moldova or Ukraine or Congo or Mongolia or Uzbekistan or Cote d’Ivoire or Morocco or Vanuatu or Djibouti or Myanmar or Vietnam or (Egypt and Arab) or Nepal or West Bank or Gaza or El Salvador or Nicaragua or Zambia or Eswatini or Nigeria or Zimbabwe or Ghana or Pakistan ) OR AB ( Angola or Honduras or Papua New Guinea or Algeria or India or Philippines or Bangladesh or Kenya or (Sao Tome and Principe) or Benin or Kiribati or Senegal or Bhutan or Kyrgyz Republic or Solomon Islands or Bolivia or Lao PDR or Sri Lanka or Cabo Verde or Lesotho or Tanzania or Cambodia or Mauritania or Timor-Leste or Cameroon or Micronesia or Tunisia or Comoros or Moldova or Ukraine or Congo or Mongolia or Uzbekistan or Cote d’Ivoire or Morocco or Vanuatu or Djibouti or Myanmar or Vietnam or (Egypt and Arab) or Nepal or West Bank or Gaza or El Salvador or Nicaragua or Zambia or Eswatini or Nigeria or Zimbabwe or Ghana or Pakistan ) 117,087

S5 ( S2 OR S3 OR S4 ) OR TI ( palliative or end of life or end-of-life or "end of life" or terminal or death or dying or mortality or incurable or futile or life-limit* or end-stage or advanced-stage* or "serious illness" or hospice* ) OR AB ( palliative or end of life or end-of-life or "end of life" or terminal or death or dying or mortality or incurable or futile or life-limit* or end-stage or advanced-stage* or "serious illness" or hospice* ) 419,329

S4 (MH "Hospices") 3,254

S3 (MH "Terminal Care+") 65,136

S2 (MH "Palliative Care") 35,990

S1 ( (personal* or individual* or household* or caregiver* or out-of-pocket* or out of pocket) N3 (cost or costs or expense or expenditure* or finance or financial or financing or incurred or payment*) ) OR ( (economic or bankruptcy or poverty) N3 (risk* or burden) ) 512,179

Web of Science 08/05/2020—1,215

# 10

1,218

#9 AND #8 AND #7

Indexes=SCI-EXPANDED, SSCI, A&HCI, CPCI-S, CPCI-SSH, BKCI-S, BKCI-SSH, ESCI, CCR-EXPANDED, IC Timespan=All years

# 9

53,078

#6 OR #5

Indexes=SCI-EXPANDED, SSCI, A&HCI, CPCI-S, CPCI-SSH, BKCI-S, BKCI-SSH, ESCI, CCR-EXPANDED, IC Timespan=All years

# 8

1,378,436

#4 OR #3 OR #2 OR #1

Indexes=SCI-EXPANDED, SSCI, A&HCI, CPCI-S, CPCI-SSH, BKCI-S, BKCI-SSH, ESCI, CCR-EXPANDED, IC Timespan=All years

# 7

3,019,856

TS=(palliative or "end of life" or end-of-life or (end* NEAR/3 life) or terminal or death or dying or mortality or incurable or futile or "life limit*" or life-limit* or "end stage" or end-stage or "advanced stage*" or advanced-stage* or "serious illness" or hospice*)

Indexes=SCI-EXPANDED, SSCI, A&HCI, CPCI-S, CPCI-SSH, BKCI-S, BKCI-SSH, ESCI, CCR-EXPANDED, IC Timespan=All years

# 6

25,446

TS=((economic or bankruptcy or poverty) NEAR/3 (risk* or burden) )

Indexes=SCI-EXPANDED, SSCI, A&HCI, CPCI-S, CPCI-SSH, BKCI-S, BKCI-SSH, ESCI, CCR-EXPANDED, IC Timespan=All years

# 5

28,630

TS=((personal* or individual* or household* or caregiver* or out-of-pocket* or "out of pocket") NEAR/3 (cost or costs or expense or expenditure* or finance or financial or financing or incurred or payment*) )

Indexes=SCI-EXPANDED, SSCI, A&HCI, CPCI-S, CPCI-SSH, BKCI-S, BKCI-SSH, ESCI, CCR-EXPANDED, IC Timespan=All years

# 4

833,368

TS=(Angola or Honduras or Papua New Guinea or Algeria or India or Philippines or Bangladesh or Kenya or (Sao Tome and Principe) or Benin or Kiribati or Senegal or Bhutan or Kyrgyz Republic or Solomon Islands or Bolivia or Lao PDR or Sri Lanka or Cabo Verde or Lesotho or Tanzania or Cambodia or Mauritania or Timor-Leste or Cameroon or Micronesia or Tunisia or Comoros or Moldova or Ukraine or Congo or Mongolia or Uzbekistan or Cote d’Ivoire or Morocco or Vanuatu or Djibouti or Myanmar or Vietnam or (Egypt and Arab) or Nepal or West Bank or Gaza or El Salvador or Nicaragua or Zambia or Eswatini or Nigeria or Zimbabwe or Ghana or Pakistan)

Indexes=SCI-EXPANDED, SSCI, A&HCI, CPCI-S, CPCI-SSH, BKCI-S, BKCI-SSH, ESCI, CCR-EXPANDED, IC Timespan=All years

# 3

384,744

TS=(Afghanistan or Guinea-Bissau or Sierra Leone or Burkina Faso or Haiti or Somalia or Burundi or Dem* People* Rep* Korea or North Korea or PRK South Sudan or Central African Republic or Liberia or Sudan or Chad or Madagascar or Syrian Arab Republic or Congo or Malawi or Tajikistan or Eritrea or Mali or Togo or Ethiopia or Mozambique or Uganda or Gambia or Niger or Yemen or Guinea or Rwanda)

Indexes=SCI-EXPANDED, SSCI, A&HCI, CPCI-S, CPCI-SSH, BKCI-S, BKCI-SSH, ESCI, CCR-EXPANDED, IC Timespan=All years

# 2

292,334

TS=((developing or "under developed" or under-developed or underdeveloped or "middle income" or "low* income" or "less* developed" or "under served" or under-served or underserved or poor* or deprived) NEAR/3 (countr* or world or population* or nation* or "gross domestic" or "gross national" or gdp or gmp or economics or economy) )

Indexes=SCI-EXPANDED, SSCI, A&HCI, CPCI-S, CPCI-SSH, BKCI-S, BKCI-SSH, ESCI, CCR-EXPANDED, IC Timespan=All years

# 1

7,358

TS=(lmic or lmics or lami countr* or lic or lics)

Indexes=SCI-EXPANDED, SSCI, A&HCI, CPCI-S, CPCI-SSH, BKCI-S, BKCI-SSH, ESCI, CCR-EXPANDED, IC Timespan=All years

Scopus, 08/05/2020—754

( TITLE-ABS-KEY ( lmic OR lmics OR lami AND countr* OR lic OR lics ) OR TITLE-ABS-KEY ( ( developing OR "under developed" OR under-developed OR underdeveloped OR "middle income" OR "low* income" OR "less* developed" OR "under served" OR under-served OR underserved OR poor* OR deprived ) W/3 ( countr* OR world OR population* OR nation* OR "gross domestic" OR "gross national" OR gdp OR gmp OR economics OR economy ) ) OR TITLE-ABS-KEY ( afghanistan OR guinea-bissau OR sierra AND leone OR burkina AND faso OR haiti OR somalia OR burundi OR dem* AND people* AND rep* AND korea OR north AND korea OR prk AND south AND sudan OR central AND african AND republic OR liberia OR sudan OR chad OR madagascar OR syrian AND arab AND republic OR congo OR malawi OR tajikistan OR eritrea OR mali OR togo OR ethiopia OR mozambique OR uganda OR gambia OR niger OR yemen OR guinea OR rwanda OR angola OR honduras OR papua AND new AND guinea OR algeria OR india OR philippines OR bangladesh OR kenya OR ( sao AND tome AND principe ) OR benin OR kiribati OR senegal OR bhutan OR kyrgyz AND republic OR solomon AND islands OR bolivia OR lao AND pdr OR sri AND lanka OR cabo AND verde OR lesotho OR tanzania OR cambodia OR mauritania OR timor-leste OR cameroon OR micronesia OR tunisia OR comoros OR moldova OR ukraine OR congo OR mongolia OR uzbekistan OR cote AND d'ivoire OR morocco OR vanuatu OR djibouti OR myanmar OR vietnam OR ( egypt AND arab ) OR nepal OR west AND bank OR gaza OR el AND salvador OR nicaragua OR zambia OR eswatini OR nigeria OR zimbabwe OR ghana OR pakistan ) ) AND ( TITLE-ABS-KEY ( ( personal* OR individual* OR household* OR caregiver* OR out-of-pocket* OR "out of pocket" ) W/3 ( cost OR costs OR expense OR expenditure* OR finance OR financial OR financing OR incurred OR payment* ) ) OR TITLE-ABS-KEY ( ( economic OR bankruptcy OR poverty ) W/3 ( risk* OR burden ) ) ) AND ( TITLE-ABS-KEY ( palliative OR "end of life" OR end-of-life OR ( end* W/3 life ) OR terminal OR death OR dying OR mortality OR incurable OR futile OR "life limit*" OR life-limit* OR "end stage" OR end-stage OR "advanced stage*" OR advanced-stage* OR "serious illness" OR hospice* ) )

EconLit (proquest), 08/05/2020— 93

(ti(lmic OR lmics OR lami countr* OR lic OR lics) OR ab(lmic OR lmics OR lami countr* OR lic OR lics) OR ((developing OR "under developed" OR under-developed OR underdeveloped OR "middle income" OR "low* income" OR "less* developed" OR "under served" OR under-served OR underserved OR poor* OR deprived) NEAR/3 (countr* OR world OR population* OR nation* OR "gross domestic" OR "gross national" OR gdp OR gmp OR economics OR economy)) OR (Afghanistan OR Guinea-Bissau OR Sierra Leone OR Burkina Faso OR Haiti OR Somalia OR Burundi OR Dem* People* Rep* Korea OR North Korea OR PRK South Sudan OR Central African Republic OR Liberia OR Sudan OR Chad OR Madagascar OR Syrian Arab Republic OR Congo OR Malawi OR Tajikistan OR Eritrea OR Mali OR Togo OR Ethiopia OR Mozambique OR Uganda OR Gambia OR Niger OR Yemen OR Guinea OR Rwanda) OR (Angola OR Honduras OR Papua New Guinea OR Algeria OR India OR Philippines OR Bangladesh OR Kenya OR (Sao Tome AND Principe) OR Benin OR Kiribati OR Senegal OR Bhutan OR Kyrgyz Republic OR Solomon Islands OR Bolivia OR Lao PDR OR Sri Lanka OR Cabo Verde OR Lesotho OR Tanzania OR Cambodia OR Mauritania OR Timor-Leste OR Cameroon OR Micronesia OR Tunisia OR Comoros OR Moldova OR Ukraine OR Congo OR Mongolia OR Uzbekistan OR Cote d’Ivoire OR Morocco OR Vanuatu OR Djibouti OR Myanmar OR Vietnam OR (Egypt AND Arab) OR Nepal OR West Bank OR Gaza OR El Salvador OR Nicaragua OR Zambia OR Eswatini OR Nigeria OR Zimbabwe OR Ghana OR Pakistan)) AND (((personal* OR individual* OR household* OR caregiver* OR out-of-pocket* OR "out of pocket") NEAR/3 (cost OR costs OR expense OR expenditure* OR finance OR financial OR financing OR incurred OR payment*)) OR ((economic OR bankruptcy OR poverty) NEAR/3 (risk* OR burden)) OR MAINSUBJECT.EXACT("Household Saving; Personal Finance (D14)")) AND (palliative OR "end of life" OR end-of-life OR (end* NEAR/3 life) OR terminal OR death OR dying OR mortality OR incurable OR futile OR "life limit*" OR life-limit* OR "end stage" OR end-stage OR "advanced stage*" OR advanced-stage* OR "serious illness" OR hospice*)

| S1 | ti(lmic or lmics or lami countr* or lic or lics) OR ab(lmic or lmics or lami countr* or lic or lics) OR ((developing or "under developed" or under-developed or underdeveloped or "middle income" or "low* income" or "less* developed" or "under served" or under-served or underserved or poor* or deprived) NEAR/3 (countr* or world or population* or nation* or "gross domestic" or "gross national" or gdp or gmp or economics or economy)) OR (Afghanistan or Guinea-Bissau or Sierra Leone or Burkina Faso or Haiti or Somalia or Burundi or Dem* People* Rep* Korea or North Korea or PRK South Sudan or Central African Republic or Liberia or Sudan or Chad or Madagascar or Syrian Arab Republic or Congo or Malawi or Tajikistan or Eritrea or Mali or Togo or Ethiopia or Mozambique or Uganda or Gambia or Niger or Yemen or Guinea or Rwanda) OR (Angola or Honduras or Papua New Guinea or Algeria or India or Philippines or Bangladesh or Kenya or (Sao Tome and Principe) or Benin or Kiribati or Senegal or Bhutan or Kyrgyz Republic or Solomon Islands or Bolivia or Lao PDR or Sri Lanka or Cabo Verde or Lesotho or Tanzania or Cambodia or Mauritania or Timor-Leste or Cameroon or Micronesia or Tunisia or Comoros or Moldova or Ukraine or Congo or Mongolia or Uzbekistan or Cote d’Ivoire or Morocco or Vanuatu or Djibouti or Myanmar or Vietnam or (Egypt and Arab) or Nepal or West Bank or Gaza or El Salvador or Nicaragua or Zambia or Eswatini or Nigeria or Zimbabwe or Ghana or Pakistan) | EconLit | 154783 |
| --- | --- | --- | --- |
| S3 | ((personal* OR individual* OR household* OR caregiver* OR out-of-pocket* OR "out of pocket") NEAR/3 (cost OR costs OR expense OR expenditure* OR finance OR financial OR financing OR incurred OR payment*)) OR ((economic OR bankruptcy OR poverty) NEAR/3 (risk* OR burden)) OR MAINSUBJECT.EXACT("Household Saving; Personal Finance (D14)") | EconLit | 23047 |
| S5 | palliative OR "end of life" OR end-of-life OR (end* NEAR/3 life) OR terminal OR death OR dying OR mortality OR incurable OR futile OR "life limit*" OR life-limit* OR "end stage" OR end-stage OR "advanced stage*" OR advanced-stage* OR "serious illness" OR hospice* | EconLit | 16384 |
| S7 | (ti(lmic OR lmics OR lami countr* OR lic OR lics) OR ab(lmic OR lmics OR lami countr* OR lic OR lics) OR ((developing OR "under developed" OR under-developed OR underdeveloped OR "middle income" OR "low* income" OR "less* developed" OR "under served" OR under-served OR underserved OR poor* OR deprived) NEAR/3 (countr* OR world OR population* OR nation* OR "gross domestic" OR "gross national" OR gdp OR gmp OR economics OR economy)) OR (Afghanistan OR Guinea-Bissau OR Sierra Leone OR Burkina Faso OR Haiti OR Somalia OR Burundi OR Dem* People* Rep* Korea OR North Korea OR PRK South Sudan OR Central African Republic OR Liberia OR Sudan OR Chad OR Madagascar OR Syrian Arab Republic OR Congo OR Malawi OR Tajikistan OR Eritrea OR Mali OR Togo OR Ethiopia OR Mozambique OR Uganda OR Gambia OR Niger OR Yemen OR Guinea OR Rwanda) OR (Angola OR Honduras OR Papua New Guinea OR Algeria OR India OR Philippines OR Bangladesh OR Kenya OR (Sao Tome AND Principe) OR Benin OR Kiribati OR Senegal OR Bhutan OR Kyrgyz Republic OR Solomon Islands OR Bolivia OR Lao PDR OR Sri Lanka OR Cabo Verde OR Lesotho OR Tanzania OR Cambodia OR Mauritania OR Timor-Leste OR Cameroon OR Micronesia OR Tunisia OR Comoros OR Moldova OR Ukraine OR Congo OR Mongolia OR Uzbekistan OR Cote d’Ivoire OR Morocco OR Vanuatu OR Djibouti OR Myanmar OR Vietnam OR (Egypt AND Arab) OR Nepal OR West Bank OR Gaza OR El Salvador OR Nicaragua OR Zambia OR Eswatini OR Nigeria OR Zimbabwe OR Ghana OR Pakistan)) AND (((personal* OR individual* OR household* OR caregiver* OR out-of-pocket* OR "out of pocket") NEAR/3 (cost OR costs OR expense OR expenditure* OR finance OR financial OR financing OR incurred OR payment*)) OR ((economic OR bankruptcy OR poverty) NEAR/3 (risk* OR burden)) OR MAINSUBJECT.EXACT("Household Saving; Personal Finance (D14)")) AND (palliative OR "end of life" OR end-of-life OR (end* NEAR/3 life) OR terminal OR death OR dying OR mortality OR incurable OR futile OR "life limit*" OR life-limit* OR "end stage" OR end-stage OR "advanced stage*" OR advanced-stage* OR "serious illness" OR hospice*) | EconLit | 93 |

Cochrane-CENTRAL (Trials) & Cochrane reviews, 08/05/2020—500

ID Search Hits

#1 lmic or lmics or lami countr* or lic or lics 664

#2 (developing or "under developed" or under-developed or underdeveloped or "middle income" or low* income or less* developed or "under served" or under-served or underserved or poor* or deprived) NEAR/3 (countr* or world or population* or nation* or "gross domestic" or "gross national" or gdp or gmp or economics or economy) 15175

#3 MeSH descriptor: [Developing Countries] explode all trees 838

#4 Afghanistan or Guinea-Bissau or Sierra Leone or Burkina Faso or Haiti or Somalia or Burundi or Dem* People* Rep* Korea or North Korea or PRK South Sudan or Central African Republic or Liberia or Sudan or Chad or Madagascar or Syrian Arab Republic or Congo or Malawi or Tajikistan or Eritrea or Mali or Togo or Ethiopia or Mozambique or Uganda or Gambia or Niger or Yemen or Guinea or Rwanda 10051

#5 Angola or Honduras or Papua New Guinea or Algeria or India or Philippines or Bangladesh or Kenya or (Sao Tome and Principe) or Benin or Kiribati or Senegal or Bhutan or Kyrgyz Republic or Solomon Islands or Bolivia or Lao PDR or Sri Lanka or Cabo Verde or Lesotho or Tanzania or Cambodia or Mauritania or Timor-Leste or Cameroon or Micronesia or Tunisia or Comoros or Moldova or Ukraine or Congo or Mongolia or Uzbekistan or Cote d'Ivoire or Morocco or Vanuatu or Djibouti or Myanmar or Vietnam or (Egypt and Arab) or Nepal or West Bank or Gaza or El Salvador or Nicaragua or Zambia or Eswatini or Nigeria or Zimbabwe or Ghana or Pakistan 45080

#6 #1 OR #2 OR #3 OR #4 OR #5 62998

#7 MeSH descriptor: [Financing, Personal] explode all trees 76

#8 MeSH descriptor: [Cost of Illness] explode all trees 807

#9 MeSH descriptor: [Costs and Cost Analysis] explode all trees 10369

#10 MeSH descriptor: [Palliative Care] explode all trees and with qualifier(s): [economics - EC] 36

#11 MeSH descriptor: [Caregivers] explode all trees and with qualifier(s): [economics - EC] 43

#12 MeSH descriptor: [Health Expenditures] explode all trees 212

#13 (personal* or individual* or household* or caregiver* or out-of-pocket* or "out of pocket") NEAR/3 (cost or costs or expense or expenditure* or finance or financial or financing or incurred or payment*) 2023

#14 (economic or bankruptcy or poverty) NEAR/3 (risk* or burden) 1580

#15 #7 OR #8 OR #9 OR #10 OR #11 OR #12 OR #13 OR #14 13296

#16 MeSH descriptor: [Palliative Care] explode all trees 1588

#17 MeSH descriptor: [Terminal Care] explode all trees 442

#18 MeSH descriptor: [Hospices] explode all trees 30

#19 MeSH descriptor: [Aged, 80 and over] explode all trees 51708

#20 palliative or "end of life" or end-of-life or (end* NEAR/3 life) or terminal or death or dying or mortality or incurable or futile or "life limit" or life-limit* or "end stage" or end-stage or "advanced stage" or advanced-stage* or "serious illness" or hospice* 154880

#21 #16 OR #17 OR #18 OR #19 OR #20 197234

#22 #6 AND #15 AND #21 in Cochrane Reviews, Trials 500
